# Letter to the Editor

**DOI:** 10.5005/jp-journals-10008-1167

**Published:** 2015-01-15

**Authors:** Syed Shoeb Ahmad

**Affiliations:** Ophthalmologist, Department of Ophthalmology Queen Elizabeth Hospital, Malaysia Phone: 00688721590 e-mail: syedshoebahmad@yahoo.com

## Postscript: An ODE to NVG

She was a girl just turned twenty-one;

A future nurse, when her course was done.

Her father has a history of DM;

But it does not cause him any problem.

The killer genes have hit her hard;

Attacking her eyes, which have no guard.

New vessels growing all over the disk;

There is also NVE, to increase the risk.

Blood vessels cover the surface of iris;

The IOP high from extensive rubeosis.

I wonder how soon she will be NPL;

Will there be TRD? A need for scleral buckle?

The retina will fight hard and bleed;

Vessels rising to the VEGF’s heed.

Macular edema is already threatening;

The posterior pole has some swelling.

The chirpy young lass does not know;

She is not prepared for the blow.

My heart goes out as I see her;

She is serene, without fear.

I reveal the fundus pictures to the family;

Shattering their cozy world completely.

‘The laser sessions will now start’;

I console her broken heart.

‘May be something will come good;

I would do, whatever I could’.

PRP shots explode on her retina;

Like bullets thundering into sandy vista.

Argon makes her cry in pain;

I need to give subtenon lignocaine.

Tears streak down her sallow cheeks;

On she comes, week after weeks.

Years pass away in a flutter;

The eyes are stable; the retinas better.

But everytime I see her in my chamber;

The questions on her lips: ‘Do I need laser?’.

Humbly, I pray to THE ONE ATOP the ladder;

‘Please make her feel and see better and better’.

